# Dichlorido{2-[(3,4-dimethyl­phen­yl)imino­meth­yl]pyridine-κ^2^ *N*,*N*′}copper(II)

**DOI:** 10.1107/S160053681104390X

**Published:** 2011-10-29

**Authors:** Mehdi Khalaj, Saeed Dehghanpour, Sadegh Salehzadeh, Ali Mahmoudi

**Affiliations:** a Department of Chemistry, Islamic Azad University, Buinzahra Branch, Qazvin, Iran; b Department of Chemistry, Alzahra University, PO Box 1993891176, Vanak, Tehran, Iran; c Faculty of Chemistry, Bu-Ali Sina University, Hamedan, Iran; d Department of Chemistry, Islamic Azad University, Karaj Branch, Karaj, Iran

## Abstract

In the title complex, [CuCl_2_(C_14_H_14_N_2_)], the Cu^II^ atom exhibits a very distorted tetra­hedral coordination geometry involving two chloride ions and two N-atom donors from the Schiff base ligand. The range for the six bond angles about the Cu^2+^ cation is 81.49 (11)–145.95 (9)°. The chelate ring including the Cu^II^ atom is approximately planar, with a maximum deviation of 0.039 (4) Å for one of the C atoms; this plane forms a dihedral angle of 46.69 (9)° with the CuCl_2_ plane.

## Related literature

For related structures, see: Mahmoudi *et al.* (2009 ▶); Wang & Zhong (2009 ▶). For background information on diimine complexes, see: Khalaj *et al.* (2010 ▶); Salehzadeh *et al.* (2011 ▶).
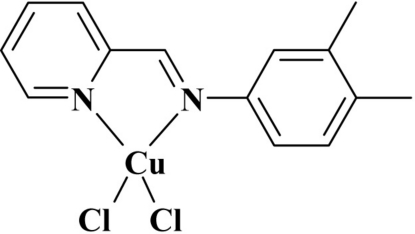


## Experimental

### 

#### Crystal data

[CuCl_2_(C_14_H_14_N_2_)]
*M* *_r_* = 344.71
Triclinic, 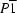

*a* = 8.1171 (4) Å
*b* = 9.5784 (4) Å
*c* = 10.0609 (5) Å
α = 67.236 (2)°
β = 88.513 (2)°
γ = 81.336 (2)°
*V* = 712.61 (6) Å^3^
*Z* = 2
Mo *K*α radiation
μ = 1.89 mm^−1^
*T* = 150 K
0.18 × 0.16 × 0.10 mm

#### Data collection

Nonius KappaCCD diffractometer
Absorption correction: multi-scan (*SORTAV*; Blessing, 1995 ▶) *T*_min_ = 0.725, *T*_max_ = 0.830
6451 measured reflections
3189 independent reflections
2256 reflections with *I* > 2σ(*I*)
*R*_int_ = 0.039

#### Refinement

*R*[*F* ^2^ > 2σ(*F* ^2^)] = 0.047
*wR*(*F* ^2^) = 0.126
*S* = 1.07
3189 reflections
174 parameters
H-atom parameters constrained
Δρ_max_ = 0.83 e Å^−3^
Δρ_min_ = −0.62 e Å^−3^

### 

Data collection: *COLLECT* (Nonius, 2002 ▶); cell refinement: *DENZO-SMN* (Otwinowski & Minor, 1997 ▶); data reduction: *DENZO-SMN*; program(s) used to solve structure: *SIR92* (Altomare *et al.*, 1994 ▶); program(s) used to refine structure: *SHELXL97* (Sheldrick, 2008 ▶); molecular graphics: *PLATON* (Spek, 2009 ▶); software used to prepare material for publication: *SHELXTL* (Sheldrick, 2008 ▶).

## Supplementary Material

Crystal structure: contains datablock(s) I, global. DOI: 10.1107/S160053681104390X/zs2154sup1.cif

Structure factors: contains datablock(s) I. DOI: 10.1107/S160053681104390X/zs2154Isup2.hkl

Additional supplementary materials:  crystallographic information; 3D view; checkCIF report

## Figures and Tables

**Table 1:** Selected bond lengths (Å)

Cu1—N1	1.988 (3)
Cu1—N2	2.025 (3)
Cu1—Cl2	2.2035 (10)
Cu1—Cl1	2.2204 (10)

**Fig. 1.**
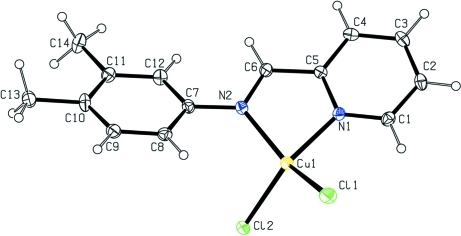
A view of the structure of the title complex, with displacement ellipsoids drawn at the 50% probability level.

## supplementary crystallographic information

### Comment

Diimine ligands derived from 2-aminopyridine and aniline derivatives are useful bidentate terminal ligands and some complexes with them as ligand have already been published (Mahmoudi *et al.*, 2009; Salehzadeh *et al.*, 2011). We report herein the crystal structure of the title complex [CuCl_2_(C_14_H_14_N_2_)] which was prepared by the reaction of CuCl_2_ with the bidentate ligand *N*-(3,4-dimethylphenyl)-pyridine-2-ylmethyleneamine.

The molecular structure of the title complex is shown in Fig. 1. The Cu^II^ ion is in a very distorted tetrahedral environment formed by a bis-chelating ligand and two Cl anions. The dihedral angle between the chelate plane Cu1–N1–C5–C6–N2 and the Cl1–Cu1–Cl2 plane is 46.69(9° and the range for the six bond angles about Cu1 is 81.49 (11)° (N1-Cu1-N2)–145.95 (9)° (N2-Cu1-Cl1). These values show an appreciable distortion towards square planar geometry. A comparison of the dihedral angles between the planes of the pyridine, chelate and the benzene rings indicate that the ligand is distorted from planarity, with a twist of 26.00 (17)° between the chelate (N1—C5—C6—N2) and the benzene (C7–C12) planes. The Cu—Cl and Cu—N bond dimensions compare well with the values found in other tetrahedral diimine complexes of copper(II) chloride (Mahmoudi *et al.*, 2009; Wang & Zhong, 2009).

### Experimental

The title complex was prepared by the reaction of CuCl_2_ (13.4 mg, 0.1 mmol) and *N*-(3,4-dimethylphenyl)-(pyridine-2-ylmethylene)amine (21.0 mg, 0.1 mmol) in 10 ml acetonitrile at room temperature. The green single crystals were obtained after the solution had been allowed to stand at room temperature for two days.

### Refinement

Hydrogen atoms were placed in calculated positions with C—H = 0.95–0.98Å and included in the refinement with *U*_iso_(H) = 1.2*U*_eq_(C_aromatic_) or 1.5*U*_eq_(C_methyl_).

### Crystal data

**Table:**

[CuCl_2_(C_14_H_14_N_2_)]	*Z* = 2
*M**_r_* = 344.71	*F*(000) = 350
Triclinic, *P*1	*D*_x_ = 1.607 Mg m^−^^3^
Hall symbol: -P 1	Mo *K*α radiation, λ = 0.71073 Å
*a* = 8.1171 (4) Å	Cell parameters from 6451 reflections
*b* = 9.5784 (4) Å	θ = 3.2–27.4°
*c* = 10.0609 (5) Å	µ = 1.89 mm^−^^1^
α = 67.236 (2)°	*T* = 150 K
β = 88.513 (2)°	Block, green
γ = 81.336 (2)°	0.18 × 0.16 × 0.10 mm
*V* = 712.61 (6) Å^3^	

### Data collection

**Table:**

Nonius KappaCCD diffractometer	3189 independent reflections
Radiation source: fine-focus sealed tube	2256 reflections with *I* > 2σ(*I*)
graphite	*R*_int_ = 0.039
Detector resolution: 9 pixels mm^-1^	θ_max_ = 27.4°, θ_min_ = 3.2°
φ scans and ω scans with κ offsets	*h* = −10→10
Absorption correction: multi-scan (*SORTAV*; Blessing, 1995)	*k* = −11→12
*T*_min_ = 0.725, *T*_max_ = 0.830	*l* = −12→13
6451 measured reflections	

### Refinement

**Table:**

Refinement on *F*^2^	Primary atom site location: structure-invariant direct methods
Least-squares matrix: full	Secondary atom site location: difference Fourier map
*R*[*F*^2^ > 2σ(*F*^2^)] = 0.047	Hydrogen site location: inferred from neighbouring sites
*wR*(*F*^2^) = 0.126	H-atom parameters constrained
*S* = 1.07	*w* = 1/[σ^2^(*F*_o_^2^) + (0.0585*P*)^2^ + 0.324*P*] where *P* = (*F*_o_^2^ + 2*F*_c_^2^)/3
3189 reflections	(Δ/σ)_max_ < 0.001
174 parameters	Δρ_max_ = 0.83 e Å^−^^3^
0 restraints	Δρ_min_ = −0.62 e Å^−^^3^

### Special details

**Table:**

Geometry. All e.s.d.'s (except the e.s.d. in the dihedral angle between two l.s. planes) are estimated using the full covariance matrix. The cell e.s.d.'s are taken into account individually in the estimation of e.s.d.'s in distances, angles and torsion angles; correlations between e.s.d.'s in cell parameters are only used when they are defined by crystal symmetry. An approximate (isotropic) treatment of cell e.s.d.'s is used for estimating e.s.d.'s involving l.s. planes.
Refinement. Refinement of *F*^2^ against ALL reflections. The weighted *R*-factor *wR* and goodness of fit *S* are based on *F*^2^, conventional *R*-factors *R* are based on *F*, with *F* set to zero for negative *F*^2^. The threshold expression of *F*^2^ > σ(*F*^2^) is used only for calculating *R*-factors(gt) *etc*. and is not relevant to the choice of reflections for refinement. *R*-factors based on *F*^2^ are statistically about twice as large as those based on *F*, and *R*- factors based on ALL data will be even larger.

### Fractional atomic coordinates and isotropic or equivalent isotropic displacement parameters (Å^2^)

**Table:**

	*x*	*y*	*z*	*U*_iso_*/*U*_eq_	
Cu1	0.78165 (5)	0.20424 (5)	0.33299 (5)	0.02388 (17)	
Cl1	1.03716 (11)	0.14201 (11)	0.26876 (12)	0.0344 (3)	
Cl2	0.82609 (12)	0.35601 (10)	0.44190 (11)	0.0285 (2)	
N1	0.7024 (4)	0.0099 (3)	0.3557 (3)	0.0214 (7)	
N2	0.5411 (4)	0.2901 (3)	0.2659 (3)	0.0210 (7)	
C1	0.7928 (5)	−0.1295 (4)	0.3991 (4)	0.0246 (8)	
H1A	0.9088	−0.1414	0.4200	0.030*	
C2	0.7211 (5)	−0.2577 (4)	0.4146 (4)	0.0268 (9)	
H2A	0.7879	−0.3557	0.4440	0.032*	
C3	0.5523 (5)	−0.2417 (4)	0.3868 (4)	0.0276 (9)	
H3A	0.5013	−0.3282	0.3973	0.033*	
C4	0.4583 (5)	−0.0967 (4)	0.3432 (4)	0.0242 (8)	
H4A	0.3416	−0.0824	0.3242	0.029*	
C5	0.5375 (4)	0.0264 (4)	0.3280 (4)	0.0199 (7)	
C6	0.4535 (5)	0.1850 (4)	0.2758 (4)	0.0226 (8)	
H6A	0.3379	0.2099	0.2500	0.027*	
C7	0.4663 (5)	0.4479 (4)	0.2081 (4)	0.0221 (8)	
C8	0.5689 (5)	0.5567 (4)	0.1391 (4)	0.0239 (8)	
H8A	0.6856	0.5271	0.1362	0.029*	
C9	0.4977 (5)	0.7090 (4)	0.0747 (4)	0.0267 (8)	
H9A	0.5672	0.7830	0.0249	0.032*	
C10	0.3303 (5)	0.7572 (4)	0.0800 (4)	0.0281 (9)	
C11	0.2260 (5)	0.6473 (4)	0.1520 (4)	0.0250 (8)	
C12	0.2963 (5)	0.4939 (4)	0.2149 (4)	0.0265 (8)	
H12A	0.2271	0.4191	0.2634	0.032*	
C13	0.2548 (5)	0.9237 (4)	0.0073 (4)	0.0321 (9)	
H13A	0.3406	0.9834	−0.0456	0.048*	
H13B	0.2103	0.9620	0.0803	0.048*	
H13C	0.1645	0.9337	−0.0601	0.048*	
C14	0.0420 (5)	0.6938 (4)	0.1616 (5)	0.0363 (10)	
H14A	−0.0082	0.6034	0.2197	0.054*	
H14B	−0.0102	0.7396	0.0644	0.054*	
H14C	0.0245	0.7687	0.2068	0.054*	

### Atomic displacement parameters (Å^2^)

**Table:**

	*U*^11^	*U*^22^	*U*^33^	*U*^12^	*U*^13^	*U*^23^
Cu1	0.0176 (3)	0.0229 (3)	0.0336 (3)	−0.00222 (18)	0.0004 (2)	−0.0139 (2)
Cl1	0.0182 (5)	0.0407 (6)	0.0514 (7)	−0.0050 (4)	0.0058 (4)	−0.0255 (5)
Cl2	0.0269 (5)	0.0226 (4)	0.0390 (6)	−0.0013 (4)	−0.0050 (4)	−0.0156 (4)
N1	0.0205 (16)	0.0209 (15)	0.0232 (17)	−0.0002 (12)	−0.0008 (13)	−0.0100 (13)
N2	0.0222 (16)	0.0190 (15)	0.0228 (16)	−0.0036 (12)	0.0033 (13)	−0.0092 (13)
C1	0.023 (2)	0.0270 (19)	0.023 (2)	0.0004 (15)	0.0001 (16)	−0.0107 (17)
C2	0.033 (2)	0.0204 (18)	0.028 (2)	−0.0005 (16)	0.0044 (18)	−0.0118 (17)
C3	0.038 (2)	0.0225 (19)	0.027 (2)	−0.0094 (17)	0.0055 (18)	−0.0130 (17)
C4	0.024 (2)	0.030 (2)	0.023 (2)	−0.0081 (16)	0.0011 (16)	−0.0129 (17)
C5	0.0194 (18)	0.0211 (17)	0.0207 (19)	−0.0042 (14)	0.0025 (15)	−0.0095 (15)
C6	0.0180 (18)	0.0235 (18)	0.027 (2)	−0.0008 (14)	0.0016 (16)	−0.0114 (17)
C7	0.026 (2)	0.0196 (17)	0.0217 (19)	−0.0018 (15)	0.0007 (16)	−0.0092 (15)
C8	0.0213 (19)	0.0262 (19)	0.026 (2)	−0.0054 (15)	0.0013 (16)	−0.0110 (17)
C9	0.031 (2)	0.0251 (19)	0.023 (2)	−0.0063 (16)	−0.0010 (17)	−0.0079 (17)
C10	0.040 (2)	0.0171 (17)	0.023 (2)	0.0025 (16)	0.0032 (18)	−0.0054 (16)
C11	0.024 (2)	0.027 (2)	0.024 (2)	−0.0012 (16)	0.0016 (16)	−0.0096 (17)
C12	0.025 (2)	0.0255 (19)	0.027 (2)	−0.0038 (16)	0.0050 (17)	−0.0090 (17)
C13	0.043 (3)	0.0233 (19)	0.029 (2)	−0.0015 (17)	−0.0030 (19)	−0.0103 (18)
C14	0.030 (2)	0.029 (2)	0.039 (3)	0.0047 (17)	0.0020 (19)	−0.0039 (19)

### Geometric parameters (Å, °)

**Table:**

Cu1—N1	1.988 (3)	C7—C8	1.391 (5)
Cu1—N2	2.025 (3)	C7—C12	1.392 (5)
Cu1—Cl2	2.2035 (10)	C8—C9	1.384 (5)
Cu1—Cl1	2.2204 (10)	C8—H8A	0.9500
N1—C1	1.335 (4)	C9—C10	1.374 (5)
N1—C5	1.348 (5)	C9—H9A	0.9500
N2—C6	1.290 (4)	C10—C11	1.413 (5)
N2—C7	1.433 (4)	C10—C13	1.510 (5)
C1—C2	1.391 (5)	C11—C12	1.390 (5)
C1—H1A	0.9500	C11—C14	1.505 (5)
C2—C3	1.379 (5)	C12—H12A	0.9500
C2—H2A	0.9500	C13—H13A	0.9800
C3—C4	1.390 (5)	C13—H13B	0.9800
C3—H3A	0.9500	C13—H13C	0.9800
C4—C5	1.383 (5)	C14—H14A	0.9800
C4—H4A	0.9500	C14—H14B	0.9800
C5—C6	1.462 (5)	C14—H14C	0.9800
C6—H6A	0.9500		
			
N1—Cu1—N2	81.49 (11)	C8—C7—C12	119.9 (3)
N1—Cu1—Cl2	145.38 (9)	C8—C7—N2	117.6 (3)
N2—Cu1—Cl2	99.33 (8)	C12—C7—N2	122.5 (3)
N1—Cu1—Cl1	95.98 (9)	C9—C8—C7	118.7 (3)
N2—Cu1—Cl1	145.95 (9)	C9—C8—H8A	120.7
Cl2—Cu1—Cl1	101.41 (4)	C7—C8—H8A	120.7
C1—N1—C5	119.2 (3)	C10—C9—C8	122.5 (3)
C1—N1—Cu1	127.1 (3)	C10—C9—H9A	118.8
C5—N1—Cu1	113.6 (2)	C8—C9—H9A	118.8
C6—N2—C7	120.0 (3)	C9—C10—C11	119.0 (3)
C6—N2—Cu1	112.6 (2)	C9—C10—C13	121.6 (3)
C7—N2—Cu1	127.4 (2)	C11—C10—C13	119.4 (3)
N1—C1—C2	121.5 (3)	C12—C11—C10	118.8 (3)
N1—C1—H1A	119.2	C12—C11—C14	120.0 (3)
C2—C1—H1A	119.2	C10—C11—C14	121.2 (3)
C3—C2—C1	119.6 (3)	C11—C12—C7	121.1 (3)
C3—C2—H2A	120.2	C11—C12—H12A	119.4
C1—C2—H2A	120.2	C7—C12—H12A	119.4
C2—C3—C4	118.8 (3)	C10—C13—H13A	109.5
C2—C3—H3A	120.6	C10—C13—H13B	109.5
C4—C3—H3A	120.6	H13A—C13—H13B	109.5
C5—C4—C3	118.8 (3)	C10—C13—H13C	109.5
C5—C4—H4A	120.6	H13A—C13—H13C	109.5
C3—C4—H4A	120.6	H13B—C13—H13C	109.5
N1—C5—C4	122.1 (3)	C11—C14—H14A	109.5
N1—C5—C6	114.1 (3)	C11—C14—H14B	109.5
C4—C5—C6	123.7 (3)	H14A—C14—H14B	109.5
N2—C6—C5	118.0 (3)	C11—C14—H14C	109.5
N2—C6—H6A	121.0	H14A—C14—H14C	109.5
C5—C6—H6A	121.0	H14B—C14—H14C	109.5
